# Association Between Diet Type and Owner‐Reported Health Conditions in Dogs in the Dog Aging Project

**DOI:** 10.1111/jvim.70060

**Published:** 2025-04-21

**Authors:** Alexandra Varela Ortiz, Ingrid Luo, Janice O'Brien, Maryanne Murphy, Angela Witzel Rollins, Matt Kaeberlein, Dog Aging Project Consortium, Audrey Ruple, Kathleen F. Kerr, M. Katherine Tolbert

**Affiliations:** ^1^ College of Veterinary Medicine, North Carolina State University Raleigh North Carolina USA; ^2^ Department of Biostatistics University of Washington Seattle Washington USA; ^3^ Department of Population Health Sciences Virginia‐Maryland College of Veterinary Medicine, Virginia Tech Blacksburg Virginia USA; ^4^ Department of Small Animal Clinical Sciences University of Tennessee Knoxville Tennessee USA; ^5^ Optispan, Inc. Seattle Washington USA; ^6^ Gastrointestinal Laboratory, Department of Small Animal Clinical Sciences School of Veterinary Medicine and Biomedical Sciences, Texas A&M University College Station Texas USA

**Keywords:** canine, extruded, home‐cooked, home‐prepared, raw

## Abstract

**Background:**

Alternative dog diets, such as home‐cooked and raw, have grown in popularity. Claims regarding health benefits for these diets have limited supporting evidence.

**Objectives:**

To evaluate whether feeding home‐cooked, commercial raw, or homemade raw diets is associated with health conditions compared to extruded diets.

**Animals:**

Twenty‐seven thousand four hundred seventy‐eight dogs.

**Methods:**

Cross‐sectional, survey‐based study. We analyzed a large cross‐sectional dataset (*n* = 27 478) of dogs fed homemade cooked (*n* = 1214), commercial raw (*n* = 961), homemade raw (*n* = 329), or extruded (*n* = 24 974) diets. We investigated associations between diet and 13 owner‐reported health condition categories. Logistic regression was used for the analysis of all health conditions.

**Results:**

Controlling for sex, age, and body size or breed, a home‐cooked diet was associated with higher odds of gastrointestinal (adjusted odds ratio (aOR): 1.4; 95% confidence interval (CI): 1.2–1.7), renal (aOR: 1.3; CI: 1.1–1.6), and hepatic disease (aOR: 1.6; CI: 1.2–2.0) compared to an extruded diet. A commercial raw diet was associated with higher odds of respiratory disease (aOR 1.7; CI: 1.3–2.3) compared to an extruded diet.

**Conclusions:**

Analysis of cross‐sectional data can only suggest effects of diet on health and are most useful for hypothesis generation or for testing existing hypotheses.

**Abbreviations**
aOR: adjusted odds ratio
DAP: Dog Aging Project
HLES: health and life experience survey

## 1 Introduction

In recent years, dog owners have become more aware of the importance of nutrition in promoting healthy aging in their dogs. As owners have taken greater interest in what they feed their pets, there is growing concern about how pet food choices contribute to nutrition‐related health outcomes [1]. This has led owners to pursue additional options for providing nutrition to their pets, including homemade cooked and raw meat‐based diets. The increasing popularity of homemade cooked diets is often attributed to distrust of commercial pet foods, the desire to feed food that resembles something the pet owner would eat, and the assumption that home‐cooked is healthier for their pets. However, when made without the close guidance of a board‐certified veterinary nutritionist, homemade cooked diets often have nutritional deficiencies [2, 3]. Raw meat‐based diets gained momentum in the 1990s and early 2000s, promoted under the idea that these diets are closer to the diets of dogs' carnivorous ancestors and are consequently healthier for dogs [4, 5]. Indeed, pet owners' interest in raw diets is often motivated by the intention to feed their animals a more natural and healthier diet [1, 4, 6]. Health benefits have been attributed to raw meat diets, including improved dental and skin health, and prevention of health disorders [7, 8]. On the other hand, there is concern from veterinarians about nutritional deficiencies in raw diets as well as the potential for food contamination and antimicrobial‐resistant enteropathogen transfer to the dog or humans in the household [9, 10, 11, 12].

Despite the increasing prevalence of raw and home‐cooked feeding practices and positive anecdotal observations from pet owners, there are limited data available to evaluate the effects of these diets on the health of dogs. The Dog Aging Project (DAP) is a large‐scale research initiative following tens of thousands of companion dogs over their lifetimes to better understand how biology, lifestyle, and environment, including diet, impact health and lifespan. Study participants are recruited to DAP through word‐of‐mouth, mainstream media, and social media. Our study objective was to use owner‐reported data from the DAP to evaluate if feeding homemade cooked, commercial raw, or homemade raw diets is associated with health conditions when compared to extruded diets.

## 2 Materials and Methods

### 2.1 Subjects

The DAP is a U.S. nationwide, long‐term longitudinal study aiming at understanding the biological and environmental determinants that affect canine health. Dog owners can register on the DAP website (https://dogagingproject.org/). Upon nominating their dogs, dog owners receive access to online portals and are invited to fill out multiple surveys throughout their participation in the DAP. While the DAP is an ongoing longitudinal study and continues to enroll new participants, the current study was a cross‐sectional analysis of dogs at study entry. The initial data set included information provided by 33 172 dog owners who joined the study by December 31, 2021, and was made public on July 7, 2022. The data were collated and managed with REDCap electronic data capture tools and are publicly accessible on the Terra platform hosted by the Broad Institute of MIT and Harvard. All data used in this report are owner‐reported. The Health and Life Experience Survey (HLES) includes eight questionnaires: Dog demographic characteristics; physical activity; environment; behavior; diet; medications, dietary supplements, and preventatives; health status; and owner demographic characteristics. Owners submitted the HLES data at the time of enrollment and were expected to provide annual updates via follow‐up surveys. The current study used information obtained from the dog demographic characteristics, owner demographic characteristics, and health status questionnaires. The University of Washington IRB deemed that the recruitment of dog owners for the DAP, and the administration and content of the DAP Health and Life Experience Survey (HLES), is human subjects research that qualifies for Category 2 exempt status (IRB ID no. 5988, effective 10/30/2018). No interactions between researchers and privately owned dogs occurred; therefore, IACUC oversight was not required. This research is based on publicly available data collected by the DAP, under U19 grant AG057377 (PI: Daniel Promislow) from the National Institute on Aging, a part of the National Institutes of Health, and by additional grants and private donations, including generous support from the Glenn Foundation for Medical Research, the Tiny Foundation Fund at Myriad Canada, the WoodNext Foundation, and the Dog Aging Institute.

Our objective was to compare owner‐reported health of dogs fed predominantly extruded diets to those fed commercial raw, homemade cooked, or homemade raw diets. Therefore, for inclusion, a dog's primary diet (comprising 51% or more of the dog's diet) needed to be one of the four diet types: extruded/dry (“extruded”), commercial raw, homemade cooked, or homemade raw. This excluded dogs whose primary diet was reported as commercial‐canned, commercial‐freeze‐dried, commercial‐semi‐moist, or other (e.g., commercially available fresh or fresh frozen diets). The age criterion was set at ≤ 18 years to capture dogs at all life stages. In addition, considering the predominant number of spayed or neutered dogs in the population and to reduce the confounding effects of hormonal fluctuations observed in intact dogs, we restricted the analysis to dogs that were either spayed or neutered. Upon applying these criteria, our analytic sample was reduced from 33 172 to 27 478 dogs.

### 2.2 Health Conditions

We studied 13 broad categories of health conditions reported in HLES: dental or oral disease, skin disorders, orthopedic disorders, gastrointestinal (GI) disorders, ear, nose, and throat disorders (ENT), renal or urinary disorders, cancer or tumors, cardiac disorders, endocrine disorders, hepatic or pancreatic disorders, neurological disorders, infectious or parasitic disorders, and respiratory disorders. Other categories of health conditions were not analyzed because they either lacked a strong justification for being diet‐related (e.g., eye disorders), they were influenced by temporary or situational factors (e.g., trauma, toxin consumption), or they were rarely reported (with fewer than 5 records for any diet type; e.g., immune‐mediated disorders, hematopoietic disorders). Within each category of health conditions, specific conditions were assessed by veterinary investigators (Alexandra Varela Ortiz, M. Katherine Tolbert) prior to data analyzes for potential associations with diet (see Table S1 for a detailed inclusion/exclusion list for health conditions). A veterinarian (Alexandra Varela Ortiz) also reviewed write‐in responses about health conditions and categorized them appropriately. Only non‐congenital conditions were considered since congenital conditions would not be affected by diet.

Our predictor of interest was the primary component of a dog's diet, defined as comprising ≥ 51% of the dog's diet according to the owner. This is a categorical variable with 4 categories: commercial‐extruded, commercial‐raw, homemade cooked, and homemade raw. A commercial‐extruded diet was the most common (91%) and was used as the reference for comparisons with the other diets (Table 1).

**TABLE 1:** Prevalence of health conditions by primary diets in 27 478 dogs that had enrolled in the Dog Aging Project by December 31, 2021.

	Extruded	Commercial raw	Home cooked	Home‐prepared raw
	*N* = 24 974	*N* = 961	*N* = 1214	*N* = 329
Health outcome—*n*, %				
Dental/oral	6377 (26%)	331 (34%)	415 (34%)	82 (25%)
Skin	6240 (25%)	261 (27%)	330 (27%)	91 (28%)
Bone/orthopedic	4440 (18%)	217 (23%)	305 (25%)	69 (21%)
Gastrointestinal	2616 (10%)	125 (13%)	188 (15%)	19 (6%)
Ear/nose/throat	1986 (8%)	75 (8%)	95 (8%)	24 (7%)
Renal/urinary	1833 (7%)	63 (7%)	135 (11%)	24 (7%)
Tumor	1675 (7%)	65 (7%)	117 (10%)	35 (11%)
Cardiac	1230 (5%)	85 (9%)	122 (10%)	14 (4%)
Brain/neurologic	830 (3%)	43 (4%)	69 (6%)	7 (2%)
Endocrine	794 (3%)	45 (5%)	61 (5%)	15 (5%)
Hepatic/pancreatic	717 (3%)	40 (4%)	80 (7%)	10 (3%)
Infection/parasites	667 (3%)	27 (3%)	30 (2%)	7 (2%)
Respiratory	564 (2%)	50 (5%)	61 (5%)	5 (2%)

### 2.3 Statistical Methods

For each health condition, we pre‐specified primary analytic model terms based on our evaluation of the relevance of age, sex, body size (as captured by weight), and breed. We adjusted for age and sex for the analysis of all 13 health conditions. Age and sex were the only adjustment variables for the analysis of ENT disorders and infectious/parasitic conditions. We additionally adjusted for body size (as measured by weight) for the analysis of dental/oral disease, skin disorders, orthopedic disorders, and respiratory disorders based on prior knowledge that the risk of a diagnosis in these categories varies with dog size. For the remaining health conditions (gastrointestinal disorders, renal or urinary disorders, cancer or tumors, cardiac disorders, endocrine disorders, neurological disorders, and hepatic or pancreas disorders), we additionally adjusted for breed in single‐breed dogs and body size (as measured by weight) in mixed‐breed dogs based on the evidence that certain breeds are known to be predisposed to these conditions.

For analyses that adjusted for breed, we only included breeds represented in at least two of the four diets in our study. This is because breeds with no variation in dietary exposure cannot inform the relationship between diet and health. We also limited analyses to breeds with at least 10 dogs meeting our inclusion criteria. Although the DAP data include over 200 breeds, our analyses that adjusted for breed included 110 breeds. Due to a striking bimodal distribution of weight among dogs reported as standard poodles, we subdivided them into large poodles (weight ≥ 13.6 kg (30 lb)) and small poodles (weight < 13.6 kg (30 lb)) and treated them as separate breeds.

Logistic regression was used for the analysis of all health conditions. Age was included in models using natural splines with knots at 2, 7, and 13 years [13]. Analyses that adjusted for weight used natural splines with knots at 15, 52, and 85 lbs. Knots are approximately at the 10th, 50th, and 90th percentile of each variable. For analyses that adjusted for breed, each breed was included as its own category; mixed‐breed dogs were included as their own category of breed, and we adjusted for weight for mixed‐breed dogs as done previously by including a MB*spline(weight) term in the regression model, where MB is a variable indicating that a dog is mixed‐breed [14]. In summary, the interpretation was comparing dogs of the same breed for purebred dogs and comparing dogs of the same weight among mixed‐breed dogs. Models adjusting for breed were fit using a conditional likelihood (with Efron approximation [15]), where the conditioning was on the breed categories. This approach allowed adjustment for breed without the possible problems from estimating over 100 nuisance parameters for breeds. We used robust standard error estimates in all analyses. Since owners must complete the entire HLES to join DAP, our study has no missing data.

Statistical analyses were carried out in R v4.2.0 using the following packages: Epi (Ns function for natural splines), survival (clogit function for conditional logistic regression), sandwich (robust standard error estimates), stats (glm function for logistic regression) and lmtest (hypothesis testing and confidence intervals).

We analyzed 13 health conditions, and for each condition, we compared dogs on each of the three non‐extruded diets to dogs on the extruded diet. This resulted in 39 hypothesis tests for our primary analysis. We used the Bonferroni correction to account for multiple comparisons, so that null hypotheses of no association were rejected if *p* < 0.0013. Since one goal of this study was to generate hypotheses about the effects of diet on dog health, results were considered suggestive if *p* < 0.05.

## 3 Results

In the analytic dataset (*n =* 27 478 dogs), 49% (*n =* 13 557 dogs) of dogs were male and 51% (*n =* 13 921 dogs) were female (Table 2). The mean (±standard deviation, SD) age was 7.3 (±4.1) years. 53% of dogs were mixed‐breed dogs (*n =* 14 691). Among single breed dogs, the most common dog breeds were Labrador retriever (6%), golden retriever (5%), and German shepherd (2%). Primary diet data showed that most dogs (91%; *n =* 24 974) were primarily fed commercially prepared extruded food. The breakdown of the remaining diet categories was as follows: homemade cooked 4% (*n =* 1214), homemade raw 1% (*n =* 321), and commercial raw 4% (*n* = 961).

**TABLE 2:** Sex, age, weight, and breed of dogs in this study.

	Extruded	Commercial raw	Homemade cooked	Homemade raw
	*N* = 24 974	*N* = 961	*N* = 1214	*N* = 329
Characteristics				
Sex—*n*, %				
Male	12 287 (49%)	484 (50%)	627 (52%)	159 (48%)
Female	12 687 (51%)	477 (50%)	587 (48%)	170 (52%)
Age—median (IQR)	7 (4, 10)	8 (5, 11)	9 (5, 12)	8 (4, 11)
Breed—*n*, %				
Single breed	11 462 (46%)	522 (54%)	593 (49%)	210 (64%)
Mixed breed	13 512 (54%)	439 (46%)	621 (51%)	119 (36%)

*Note:* When analytic models adjusted for breed, different breeds were kept separate but reported together as ‘single breed’ here.

The prevalence of health conditions according to primary diet are reported in Table 1. In the analytic dataset, 17 393 dogs (63%) presented with at least one of the 13 health conditions potentially related to diet. Of those, dental/oral disease was the most common owner‐reported health condition, affecting 7204 or 26% of dogs (Table 1). Dental calculus was the most commonly reported dental condition with 4147 dogs being reported by their owners with this condition. Skin disease was the second most common owner‐reported health condition, affecting 6922 or 25% of dogs. Seasonal allergy was the most commonly reported skin health condition with 1931 dogs affected.

**FIGURE 1:**
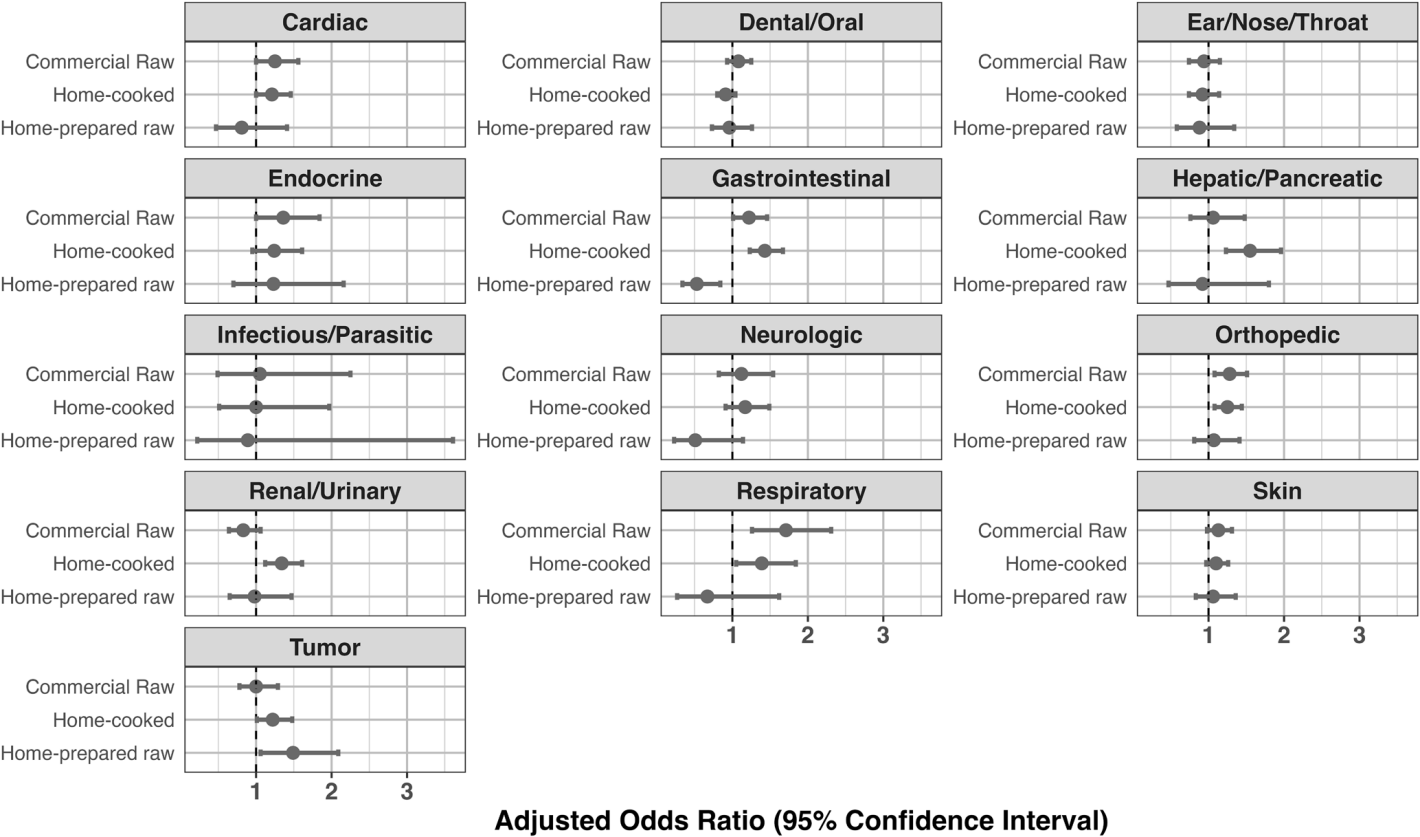
Adjusted odds ratios with 95% confidence intervals for association between each of 13 disease categories and diet type compared to extruded diets. Using Bonferroni correction for 39 tests (*p* < 0.0013), statistically significant associations were identified for respiratory disease and commercial raw diets, as well as GI, hepatic/pancreatic, and renal/urinary disease and homemade cooked diet.

With Bonferroni correction, we found statistically significant associations between diet and the presence of a health condition for four out of the 13 health condition categories (Figure 1 and Table 3). Controlling for sex, age, body size (for mixed‐breed dogs), and breed (for single‐breed dogs), dogs fed a home‐cooked diet were more likely to have gastrointestinal (adjusted odds ratio (aOR); 95% confidence interval (CI): 1.4; 1.2–1.7), renal/urinary (aOR: 1.3; CI: 1.1–1.6), and hepatic/pancreatic disease (aOR: 1.6; CI: 1.2–2.0) compared to dogs fed extruded diets (Figure 1A). Dogs fed a commercial raw diet were more likely to have respiratory disease (OR 1.7; CI: 1.3–2.3; Figure 1B; *p* ≤ 0.001 for all). For the four statistically significant findings, results are similar with different adjustment models (Table S2). However, we note that the pre‐specified analytic model for respiratory conditions did not adjust for breed and, if adjusting for breed, the observed association is attenuated and no longer statistically significant with the Bonferroni p‐value threshold (Table S2). No other associations between diet and health conditions were statistically significant using the Bonferroni *p*‐value threshold, although eight associations were suggestive (*p* < 0.05) as shown in Table 3.

**TABLE 3:** Analytic results for each health condition. Adjusted odds ratios (aOR) compare each diet to an extruded diet.

Adjustment model: Age and sex		
	aOR (95% CI)	*p*
Ear/nose/throat		
Commercial raw	0.94 (0.74–1.19)	0.5974
Home‐cooked	0.92 (0.74–1.14)	0.4448
Home‐prepared raw	0.88 (0.58–1.34)	0.5634
Infection/parasites		
Commercial raw	1.05 (0.49–2.25)	0.9093
Home‐cooked	1.00 (0.51–1.97)	0.9986
Home‐prepared raw	0.89 (0.22–3.61)	0.8725

Adjustment model: Age, sex, and weight		
Dental/oral		
Commercial raw	1.08 (0.93–1.25)	0.3096
Home‐cooked	0.91 (0.80–1.04)	0.1858
Home‐prepared raw	0.96 (0.73–1.26)	0.7562
Skin		
Commercial raw	1.13 (0.98–1.31)	0.0971
Home‐cooked	1.10 (0.97–1.26)	0.1478
Home‐prepared raw	1.06 (0.83–1.36)	0.6228
Bone/orthopedic		
Commercial raw	1.28 (1.08–1.51)	0.0046*
Home‐cooked	1.25 (1.08–1.44)	0.0033
Home‐prepared raw	1.07 (0.81–1.41)	0.6339
Respiratory		
Commercial raw	**1.71 (1.26–2.31)**	**0.0006**
Home‐cooked	1.39 (1.05–1.84)	0.0203*
Home‐prepared raw	0.67 (0.27–1.62)	0.3699

Adjustment model: Age, sex, weight or breed		
Gastrointestinal		
Commercial raw	1.22 (1.01–1.46)	0.0373*
Home‐cooked	**1.43 (1.23–1.67)**	**< 0.0001**
Home‐prepared raw	0.53 (0.34–0.84)	0.0064*
Renal/urinary		
Commercial raw	0.83 (0.64–1.06)	0.1372
Home‐cooked	**1.34 (1.12–1.61)**	**0.0012**
Home‐prepared raw	0.98 (0.65–1.47)	0.9295
Tumor		
Commercial raw	1.00 (0.78–1.29)	0.9945
Home‐cooked	1.22 (1.01–1.48)	0.0359*
Home‐prepared raw	1.49 (1.06–2.09)	0.0223*
Cardiac		
Commercial raw	1.25 (1.00–1.56)	0.0474*
Home‐cooked	1.21 (1.00–1.46)	0.0547
Home‐prepared raw	0.81 (0.47–1.41)	0.4521
Brain/neurologic		
Commercial raw	1.12 (0.82–1.54)	0.4754
Home‐cooked	1.17 (0.91–1.49)	0.221
Home‐prepared raw	0.51 (0.23–1.14)	0.1003
Endocrine		
commercial raw	1.36 (1.00–1.84)	0.0499*
Home‐cooked	1.24 (0.95–1.61)	0.1105
Home‐prepared raw	1.23 (0.70–2.16)	0.4627
Hepatic/pancreatic		
Commercial raw	1.06 (0.76–1.48)	0.732
Home‐cooked	**1.55 (1.23–1.96)**	**0.0002**
Home‐prepared raw	0.92 (0.47–1.80)	0.8122

*Note:* Associations that are statistically significant after Bonferroni correction for 39 tests are shown in bold, and suggestive associations are noted with an asterisk (*).

For the four categories of health conditions that were significantly associated with a non‐extruded diet, we note the most common diagnoses within each category. The most common GI condition was chronic or recurrent diarrhea (*n* = 750, or 25% of dogs with a GI condition). The most common hepatic/pancreatic condition was pancreatitis (*n* = 534, or 63% of dogs with a hepatic/pancreatic condition). The most common renal/urinary condition was recurrent urinary tract infection (*n* = 804 dogs, or 37% of dogs with a renal/urinary condition). Chronic cough was the most commonly reported respiratory condition (*n* = 224 dogs; 33% of dogs with a respiratory condition).

## 4 Discussion

There is growing interest in the role of nutrition in canine health. Over the past few years, a few studies have evaluated the association of diet type on canine health. In a prospective study in which biochemical and veterinarian‐assisted clinical health outcomes were evaluated in dogs fed extruded or commercially prepared raw meat‐based diets, there was a slight improvement in clinical health scores in dogs fed the raw meat‐based diets. However, the study was relatively small (55 dogs), did not control for breed or sex, and only targeted specific dental and integumentary health outcomes [16]. Aside from our current study, there are only a handful of other large (i.e., > 500 dogs) studies that evaluated diet type and health outcomes in dogs. The prior studies focused solely on the role of early life diet patterns and the development of otitis, atopic dermatitis, or gastrointestinal disease in Finnish dogs [7, 8, 17, 18]. Using data from over 27 000 dogs from the DAP, this is the largest published study to evaluate the association of diet type and a variety of owner‐reported health conditions in dogs across a wide range of ages.

A dental or oral condition was the most common health condition reported in our enrolled dogs. There was no association between diet type and dental/oral health conditions. An extruded diet, especially one with a larger kibble size to increase prehension and mastication, has been demonstrated to reduce plaque deposits in several studies of dogs [19, 20]. However, these studies were based on veterinarian examinations whereas our findings are based on owner‐reported health information. Future studies using medical record data obtained from the DAP dogs would help to determine if veterinarian assessments would identify a benefit to feeding an extruded diet on the reduction of plaque deposits or other dental diseases. Owners might also choose different diet types following the development of oral or dental conditions. Thus, future studies should use epidemiological methods to estimate the effect of dental disease on the decisions of owners to change diet type.

A skin condition was the second most common health condition in this study. As with dental or oral conditions, we did not find an association with diet type and owner‐reported skin health condition. This contrasts with two large cross‐sectional studies in which investigators identified a lower risk of owner‐reported atopic dermatitis in dogs being fed raw food for a portion of their diet (≥ 20%) compared to dogs solely fed an extruded diet [7, 18]. In these studies, investigators evaluated the influence of early life exposure to raw feeding on the development of atopic dermatitis later in life. Both allergy and non‐allergy prone breeds were evaluated, but it does not appear that an adjustment was made to control for breed when comparing diet types. Like the current study, both studies relied solely on owner‐reported health data. A notable difference is that our study examines a variety of possible cutaneous health conditions across a wide range of dog ages. In another study, investigators also identified an improvement in veterinarian‐evaluated skin health in raw‐fed dogs compared to dogs fed extruded diets; however, there was no difference in the prevalence of previous skin disease as reported by the owner between raw‐fed dogs and dogs fed an extruded, kibble diet [16].

The benefit of early life exposure to raw foods in dogs has been suggested in a previous questionnaire‐based study in which dogs with a raw feeding exposure in early life were less likely to have inflammatory bowel disease compared to dogs fed an extruded diet; however, the study did not appear to control for breed and used different age cutoffs for case versus control dogs [8]. Results in the current study do not support such an association. In fact, in the current study, dogs fed a commercial raw diet were more likely to have a GI health condition than dogs fed an extruded diet, but the observed association was weak and not statistically significant using the Bonferroni‐adjusted threshold (aOR: 1.2; CI: 1.0–1.5; *p* = 0.037). On the other hand, dogs fed a homemade raw diet appeared less likely to have a GI health condition compared to an extruded diet; however, this difference was not statistically significant (aOR: 0.5; CI: 0.3–0.8; *p* = 0.006) using the Bonferrnoi threshold.

Dogs fed a home‐cooked diet were also more likely to have an owner‐reported gastrointestinal condition. The reason for this association is unclear, but as home‐cooking is a strategy that is often employed for the treatment of gastrointestinal disease, it is possible this finding is the result of a reverse causal association. This also might be the case for an increase in the prevalence of hepatic/pancreatic and renal/urinary disease in dogs being fed a homemade cooked diet.

In our study, dogs fed primarily a commercial raw diet were more likely to have an owner‐reported respiratory health condition. There are a few possible reasons for this finding. In a study of dogs in the DAP [21], dogs fed raw diets were more likely to be reported by owners as working or agility dogs and were more likely to be part of multi‐dog households. Thus, it is possible that these dogs have a higher risk for communicable respiratory infections as a result of their environments. Raw diets are also typically higher in dietary fat compared to extruded diets [11]. High dietary fat can slow gastric emptying [22] and decrease lower esophageal sphincter pressure [23], so it is possible that raw food diets increase gastroesophageal reflux and pulmonary aspiration in at‐risk dogs. An additional consideration is that owners of dogs with respiratory diseases might turn to feeding commercial raw diets following the development of a respiratory condition due to a perception that this diet type will be a healthier option for their dog. Finally, we note that the analytic model for respiratory conditions did not adjust for breed because the specific conditions in this category are not known to be more or less common in individual breeds. However, we acknowledge that the observed association is weaker in a model that adjusts for breed (Table S2).

We found no statistically significant increase in infectious or parasitic conditions among dogs consuming commercial or homemade raw diets, even though the category included foodborne infections like salmonellosis. The relatively low frequency of microbial investigations in veterinary practice for foodborne infections might have led to these infections being underreported or misclassified as general gastrointestinal (GI) conditions. However, we also did not find statistically significant associations between raw diets and GI conditions. On the other hand, there was a suggestive positive association between commercial raw diets and GI conditions and a suggestive negative association between home‐prepared raw diets and GI conditions. The latter suggestive association is stronger but might represent reverse causation.

Analysis of this large cross‐sectional dataset identified additional associations that were suggestive but were not statistically significant according to the Bonferroni *p*‐value threshold. These include suggestive positive associations between a commercial raw diet and endocrine, cardiac, and bone/orthopedic conditions, as well as suggestive positive associations between both home‐cooked and home‐prepared raw diets and tumor conditions. These data might suggest valuable directions for future longitudinal studies that can more rigorously examine possible causal relationships between alternative diets and health.

This study is a cross‐sectional analysis, examining the association between diet and health conditions at a single time point. The key study limitation is that owners might have selected their dog's diet as a way of managing a disease, meaning the disease led to the diet and not the other way around. This issue of “reverse causation” is of particular concern for gastrointestinal conditions since dietary modification can play an important role in managing these conditions. Another limitation is that all data are owner‐reported and thus subject to error in recall and interpretation. Although a veterinarian reviewed the owner's responses, there could still be differences in interpretation. We also lacked detailed data about the composition of our diets of interest. There is great variability in both nutrient and ingredient content among different diets in the same category. Additionally, this study did not examine dogs being fed a commercial fresh or fresh‐frozen diet. The DAP attempts to recruit dogs with owners who represent the diversity of dog owners in the United States. However, the DAP study population is acknowledged to overrepresent owners with high socioeconomic and education statuses. Finally, due to the limited number of intact dogs in our sample, analyzes included only spayed and neutered dogs. While age at spay/neuter might be an important factor for some health conditions, this information was not used in our analysis because data on the timing of gonadectomy was not sufficiently detailed.

As the DAP continues to collect longitudinal data from owner surveys and from medical record data, investigators can prospectively examine dogs with different types of diets and see if certain health conditions are more likely to develop in dogs with particular diets. Such prospective studies provide stronger evidence for causal effects of diet on canine health.

## Disclosure

Authors declare no off–label use of antimicrobials.

## Ethics Statement

No interactions between researchers and privately owned dogs occurred; therefore, Institutional Animal Care and Use Committee oversight was not required. The University of Washington Institutional Review Board (IRB) deemed that recruitment of dog owners for the Dog Aging Project (DAP), and the administration and content of the DAP Health and Life Experience Survey (HLES), are human subjects research that qualifies for Category 2 exempt status (IRB ID no. 5988, effective 10/30/2018).

## Conflicts of Interest

The authors declare no conflicts of interest.

## Supporting information


**Table S1.** Specific exclusions and inclusions for health condition categories. Italics represent conditions in which < 100 dogs were reported.
**Table S2.** Analytic results for each health condition with different adjustment models. Adjusted odds ratios (aOR) compare each diet to a standard extruded diet (kibble). For each health condition, the pre‐specified adjustment model is shaded and, within these, results that are statistically significant using the Bonferroni *p*‐value threshold 0.0013 are shown in bold.
**Table S3.** Logistic regression results for pre‐specified analytic models for all 13 health conditions. *Age was modeled using natural splines with knots at 2, 7, and 13 years **Weight was modeled using natural splines with knots at 15, 52, and 85 lbs. ***Models were fit with conditional logistic regression which do not provide estimates for breed effects.
