# Supplementary material for: Association Between Diet Type and Owner‐Reported Health Conditions in Dogs in the Dog Aging Project
Source: J Vet Intern Med. 2025 Apr 21;39(3):e70060. doi: 10.1111/jvim.70060 (PMC12010193; doi:10.1111/jvim.70060)
Supplement: Supplementary file 1 — Table S1. Specific exclusions and inclusions for health condition categories. Italics represent conditions in which < 100 dogs were reported. Table S2. Analytic results for each health condition with different adjustment models. Adjusted odds ratios (aOR) compare each diet to a standard extruded diet (kibble). For each health condition, the pre‐specified adjustment model is shaded and, within these, results that are statistically significant using the Bonferroni p‐value threshold 0.0013 are shown in bold. Table S3. Logistic regression results for pre‐specified analytic models for all 13 health conditions. *Age was modeled using natural splines with knots at 2, 7, and 13 years **Weight was modeled using natural splines with knots at 15, 52, and 85 lbs. ***Models were fit with conditional logistic regression which do not provide estimates for breed effects. [file JVIM-39-e70060-s001.docx]

| **Health condition category** | **Specific Exclusions** | **Specific Inclusions** |
| --- | --- | --- |
| **Dental/Oral** | Underbite, retained deciduous, overbite | Dental calculus, extracted teeth, fractured teeth, gingivitis, *oronasal fistula*, *sialocele* |
| **Skin** | Ectoparasites (fleas, ticks), flea allergy dermatitis | Seasonal allergies, pruritus, sebaceous cysts, allergies that affect the skin, chronic or recurrent hot spots, atopic dermatitis, alopecia, non-specific dermatosis, lick granuloma, pyoderma, mange, *seborrhea, pododermatitis, discoid lupus, ichthyosis, sebaceous adenitis, systemic lupus erythematosus, panepidermal pustular pemphigus foliaceus, erythematosus, or vulgaris* |
| **Bone/Orthopedic** | *Dwarfism, osteomyelitis* | Osteoarthritis, cruciate ligament rupture, hip dysplasia, patellar luxation, lameness, degenerative joint disease, intervertebral disc disease, elbow dysplasia, spondylosis, *growth deformity, osteochondritis dissecans, panosteitis, carpal subluxation syndrome* |
| **Gastrointestinal** | Foreign body ingestion or blockage, *pyloric stenosis, megaesophagus* | Chronic or recurrent diarrhea, anal sac impaction, food or medicine allergies, chronic or recurrent vomiting, stress colitis or hemorrhagic gastroenteritis, other GI allergies, bilious vomiting syndrome, *fecal incontinence*, *constipation, bloat with torsion, idiopathic canine colitis, protein-losing enteropathy, lymphangiectasia, malabsorptive disorder* |
| **Ear/Nose/Throat** | *Epistaxis*, hematoma, ear mites, hearing loss, deafness | Otitis, *pharyngitis*, *tonsilitis* |
| **Renal/Urinary** | *Ectopic ureter, bladder prolapse, renal dysplasia, urethral prolapse, tubular disorder* | Urinary tract infection, urinary incontinence, urinary crystals or uroliths, chronic kidney disease, *proteinuria, acute kidney injury, kidney stones, pyelonephritis* |
| **Tumor/Cancer** | None | All tumor types included |
| **Cardiac** | *Subaortic stenosis, pulmonic stenosis* | Murmur, congestive heart failure, valve disease, arrhythmia, *cardiomyopathy*, *hypertension, pulmonary hypertension, other cardiac, endocarditis, pericardial effusion* |
| **Hepatic/Pancreatic** | *Gall bladder surgery, biliary obstruction, portosystemic shunt, gall bladder rupture, microvascular dysplasia* | Pancreatitis, *chronic inflammatory liver disorder, exocrine pancreatic insufficiency*, *gall bladder mucocele* |
| **Brain/Neurologic** | Vestibular disease, *laryngeal paralysis, limb paralysis, horner’s syndrome, polyneuropathy, wobbler syndrome, fibrocartilaginous embolism, cauda equina syndrome, diskospondylitis, myasthenia gravis* | Seizures, intervertebral disc disease, senility or dementia, *degenerative myelopathy* |
| **Infection/Parasites** | Tapeworms, roundworms, hookworms, lyme disease, coccidia, whipworms, parvovirus, heartworm infection, *dermatophytosis*, *leptospirosis, influenza, fungal infection, tickborne infection, yersinia pestis, tularemia* | GI parasites, *fever of unknown origin, staphylococcus, campylobacteriosis, salmonellosis* |
| **Respiratory** | Laryngeal paralysis, *tracheal stenosis, elongated soft palate,* *stenotic nares*, *acute respiratory distress syndrome, pulmonary bullae, long lobe torsion* | Chronic or recurrent cough, pneumonia, tracheal collapse, *chronic or recurrent bronchitis*, *chronic or recurrent rhinitis* |

*Supplementary Table 1.* Specific exclusions and inclusions for health condition categories. Italics represent conditions in which < 100 dogs were reported.

|  | **Adjustment Model** | | | | |  |
| --- | --- | --- | --- | --- | --- | --- |
|  | age and sex | | age, sex, weight | | age, sex, and weight or breed | |
|  | OR (95% CI) | P value | OR (95% CI) | P value | OR (95% CI) | P value |
| Ear/Nose/Throat |  |  |  |  |  |  |
| Commercial Raw | 0.94 (0.74-1.19) | 0.5974 | 1.06 (0.83-1.35) | 0.6433 | 1.04 (0.83-1.32) | 0.7131 |
| Home-cooked | 0.92 (0.74-1.14) | 0.4448 | 1.03 (0.83-1.28) | 0.7857 | 1.03 (0.84-1.27) | 0.7841 |
| Home-prepared Raw | 0.88 (0.58-1.34) | 0.5634 | 0.82 (0.54-1.25) | 0.3567 | 0.95 (0.63-1.42) | 0.7923 |
| Infection/Parasites |  |  |  |  |  |  |
| Commercial Raw | 1.05 (0.49-2.25) | 0.9093 | 1.18 (0.54-2.55) | 0.6792 | 1.01 (0.44-2.31) | 0.9853 |
| Home-cooked | 1.00 (0.51-1.97) | 0.9986 | 1.12 (0.57-2.22) | 0.7471 | 1.13 (0.57-2.23) | 0.7332 |
| Home-prepared Raw | 0.89 (0.22-3.61) | 0.8725 | 0.85 (0.21-3.43) | 0.8165 | 1.01 (0.24-4.23) | 0.989 |
| Dental/Oral |  |  |  |  |  |  |
| Commercial Raw | 1.36 (1.18-1.57) | < 0.0001 | 1.08 (0.93-1.25) | 0.3096 | 1.08 (0.97-1.20) | 0.1625 |
| Home-cooked | 1.15 (1.00-1.31) | 0.0432 | 0.91 (0.80-1.04) | 0.1858 | 0.94 (0.86-1.04) | 0.2187 |
| Home-prepared Raw | 0.85 (0.65-1.12) | 0.2486 | 0.96 (0.73-1.26) | 0.7562 | 0.94 (0.75-1.17) | 0.5922 |
| Skin |  |  |  |  |  |  |
| Commercial Raw | 1.06 (0.91-1.22) | 0.463 | 1.13 (0.98-1.31) | 0.0971 | 1.08 (0.96-1.23) | 0.2031 |
| Home-cooked | 1.03 (0.90-1.18) | 0.6555 | 1.10 (0.97-1.26) | 0.1478 | 1.07 (0.96-1.20) | 0.209 |
| Home-prepared Raw | 1.10 (0.86-1.41) | 0.44 | 1.06 (0.83-1.36) | 0.6228 | 1.09 (0.89-1.35) | 0.3956 |
| Bone/Orthopedic |  |  |  |  |  |  |
| Commercial Raw | 1.17 (0.99-1.39) | 0.0611 | 1.28 (1.08-1.51) | 0.0046 | 1.19 (1.03-1.37) | 0.0156 |
| Home-cooked | 1.16 (1.00-1.34) | 0.0433 | 1.25 (1.08-1.44) | 0.0033 | 1.16 (1.03-1.30) | 0.0171 |
| Home-prepared Raw | 1.13 (0.86-1.49) | 0.3844 | 1.07 (0.81-1.41) | 0.6339 | 1.04 (0.81-1.32) | 0.776 |
| Respiratory |  |  |  |  |  |  |
| Commercial Raw | 2.08 (1.54-2.81) | < 0.0001 | **1.71 (1.26-2.31)** | **0.0006** | 1.58 (1.17-2.13) | 0.0029 |
| Home-cooked | 1.67 (1.27-2.21) | 0.0003 | 1.39 (1.05-1.84) | 0.0203 | 1.35 (1.04-1.77) | 0.0268 |
| Home-prepared Raw | 0.61 (0.25-1.49) | 0.2806 | 0.67 (0.27-1.62) | 0.3699 | 0.68 (0.28-1.66) | 0.3945 |
| Gastrointestinal |  |  |  |  |  |  |
| Commercial Raw | 1.22 (1.01-1.49) | 0.0411 | 1.25 (1.03-1.52) | 0.0233 | 1.22 (1.01-1.46) | 0.0373 |
| Home-cooked | 1.45 (1.23-1.70) | < 0.0001 | 1.48 (1.25-1.74) | < 0.0001 | **1.43 (1.23-1.67)** | **< 0.0001** |
| Home-prepared Raw | 0.51 (0.32-0.81) | 0.0044 | 0.50 (0.32-0.80) | 0.0037 | 0.53 (0.34-0.84) | 0.0064 |
| Renal/Urinary |  |  |  |  |  |  |
| Commercial Raw | 0.80 (0.62-1.04) | 0.0929 | 0.83 (0.64-1.08) | 0.163 | 0.83 (0.64-1.06) | 0.1372 |
| Home-cooked | 1.30 (1.07-1.57) | 0.0083 | 1.34 (1.10-1.63) | 0.0034 | **1.34 (1.12-1.61)** | **0.0012** |
| Home-prepared Raw | 0.92 (0.60-1.41) | 0.7082 | 0.90 (0.59-1.38) | 0.6331 | 0.98 (0.65-1.47) | 0.9295 |
| Tumor |  |  |  |  |  |  |
| Commercial Raw | 0.84 (0.65-1.10) | 0.2074 | 1.02 (0.78-1.33) | 0.8952 | 1.00 (0.78-1.29) | 0.9945 |
| Home-cooked | 1.06 (0.87-1.30) | 0.5617 | 1.25 (1.01-1.53) | 0.0377 | 1.22 (1.01-1.48) | 0.0359 |
| Home-prepared Raw | 1.53 (1.06-2.21) | 0.0223 | 1.44 (0.99-2.08) | 0.0544 | 1.49 (1.06-2.09) | 0.0223 |
| Cardiac |  |  |  |  |  |  |
| Commercial Raw | 1.59 (1.26-2.02) | 0.0001 | 1.29 (1.01-1.65) | 0.0427 | 1.25 (1.00-1.56) | 0.0474 |
| Home-cooked | 1.48 (1.21-1.82) | 0.0002 | 1.24 (1.00-1.52) | 0.0459 | 1.21 (1.00-1.46) | 0.0547 |
| Home-prepared Raw | 0.76 (0.44-1.32) | 0.3334 | 0.84 (0.49-1.46) | 0.5412 | 0.81 (0.47-1.41) | 0.4521 |
| Brain/Neurologic |  |  |  |  |  |  |
| Commercial Raw | 1.14 (0.83-1.58) | 0.4072 | 1.09 (0.79-1.52) | 0.5874 | 1.12 (0.82-1.54) | 0.4754 |
| Home-cooked | 1.22 (0.95-1.58) | 0.1229 | 1.17 (0.91-1.52) | 0.2242 | 1.17 (0.91-1.49) | 0.221 |
| Home-prepared Raw | 0.57 (0.27-1.20) | 0.1374 | 0.57 (0.27-1.22) | 0.1471 | 0.51 (0.23-1.14) | 0.1003 |
| Endocrine |  |  |  |  |  |  |
| Commercial Raw | 1.26 (0.92-1.72) | 0.1489 | 1.40 (1.02-1.92) | 0.0372 | 1.36 (1.00-1.84) | 0.0499 |
| Home-cooked | 1.14 (0.87-1.49) | 0.3561 | 1.23 (0.94-1.62) | 0.1333 | 1.24 (0.95-1.61) | 0.1105 |
| Home-prepared Raw | 1.32 (0.77-2.25) | 0.3078 | 1.26 (0.74-2.15) | 0.3939 | 1.23 (0.70-2.16) | 0.4627 |
| Hepatic/Pancreatic |  |  |  |  |  |  |
| Commercial Raw | 1.26 (0.91-1.74) | 0.1728 | 1.09 (0.78-1.51) | 0.6271 | 1.06 (0.76-1.48) | 0.732 |
| Home-cooked | 1.73 (1.35-2.21) | < 0.0001 | 1.53 (1.20-1.96) | 0.0007 | **1.55 (1.23-1.96)** | **0.0002** |
| Home-prepared Raw | 0.96 (0.51-1.81) | 0.9037 | 1.03 (0.54-1.94) | 0.9314 | 0.92 (0.47-1.80) | 0.8122 |

*Supplementary Table 2.* Analytic results for each health condition with different adjustment models. Adjusted odds ratios (aOR) compare each diet to a standard extruded diet (kibble). For each health condition, the pre-specified adjustment model is shaded and, within these, results that are statistically significant using the Bonferroni P-value threshold 0.0013 are shown in bold.

| Adjustment Model: | age* and sex |  |  |
| --- | --- | --- | --- |
|  | **Beta (95% CI)** | **df** | **P value** |
| **Ear/Nose/Throat** |  |  |  |
| Commercial Raw | -0.06 (-0.30-0.18) | 1 | 0.5974 |
| Home-cooked | -0.08 (-0.30-0.13) | 1 | 0.4448 |
| Home-prepared Raw | -0.12 (-0.54-0.30) | 1 | 0.5634 |
| Age (natural spline basis 1) | 1.01 (0.79-1.23) | 2 | < 0.0001 |
| Age (natural spline basis 2) | 0.37 (0.27-0.48) |  |  |
| Female | -0.20 (-0.29--0.11) | 1 | < 0.0001 |
| **Infection/Parasites** |  |  |  |
| Commercial Raw | 0.04 (-0.72-0.81) | 1 | 0.9093 |
| Home-cooked | 0.00 (-0.68-0.68) | 1 | 0.9986 |
| Home-prepared Raw | -0.11 (-1.51-1.28) | 1 | 0.8725 |
| Age (natural spline basis 1) | 0.45 (-0.26-1.15) | 2 | 0.4488 |
| Age (natural spline basis 2) | 0.52 (0.19-0.86) |  |  |
| Female | -0.06 (-0.34-0.23) | 1 | 0.7087 |
|  |  |  |  |
| Adjustment Model: | age*, sex, and weight** |  |  |
|  | **Beta (95% CI)** | **df** | **P value** |
| **Dental/Oral** |  |  |  |
| Commercial Raw | 0.08 (-0.07-0.23) | 1 | 0.3096 |
| Home-cooked | -0.09 (-0.22-0.04) | 1 | 0.1858 |
| Home-prepared Raw | -0.04 (-0.32-0.23) | 1 | 0.7562 |
| Age (natural spline basis 1) | 4.44 (4.24-4.64) | 2 | < 0.0001 |
| Age (natural spline basis 2) | 1.87 (1.80-1.95) |  |  |
| Female | -0.09 (-0.15--0.03) | 1 | 0.0052 |
| Weight (natural spline basis 1) | -2.01 (-2.14--1.87) | 2 | < 0.0001 |
| Weight (natural spline basis 2) | -0.81 (-0.88--0.74) |  |  |
| **Skin** |  |  |  |
| Commercial Raw | 0.12 (-0.02-0.27) | 1 | 0.0971 |
| Home-cooked | 0.10 (-0.03-0.23) | 1 | 0.1478 |
| Home-prepared Raw | 0.06 (-0.18-0.31) | 1 | 0.6228 |
| Age (natural spline basis 1) | 1.43 (1.28-1.57) | 2 | < 0.0001 |
| Age (natural spline basis 2) | 0.57 (0.50-0.63) |  |  |
| Female | -0.10 (-0.16--0.05) | 1 | 0.0002 |
| Weight (natural spline basis 1) | 0.58 (0.46-0.71) | 2 | < 0.0001 |
| Weight (natural spline basis 2) | 0.28 (0.23-0.34) |  |  |
| **Bone/Orthopedic** |  |  |  |
| Commercial Raw | 0.25 (0.08-0.41) | 1 | 0.0046* |
| Home-cooked | 0.22 (0.07-0.37) | 1 | 0.0033 |
| Home-prepared Raw | 0.07 (-0.21-0.34) | 1 | 0.6339 |
| Age (natural spline basis 1) | 3.28 (3.06-3.50) | 2 | < 0.0001 |
| Age (natural spline basis 2) | 1.92 (1.84-2.00) |  |  |
| Female | 0.06 (-0.01-0.13) | 1 | 0.0723 |
| Weight (natural spline basis 1) | 0.57 (0.41-0.73) | 2 | < 0.0001 |
| Weight (natural spline basis 2) | 0.50 (0.44-0.56) |  |  |
| **Respiratory** |  |  |  |
| Commercial Raw | **0.53 (0.23-0.84)** | **1** | **0.0006** |
| Home-cooked | 0.33 (0.05-0.61) | 1 | 0.0203* |
| Home-prepared Raw | -0.41 (-1.30-0.48) | 1 | 0.3699 |
| Age (natural spline basis 1) | 1.72 (1.23-2.21) | 2 | < 0.0001 |
| Age (natural spline basis 2) | 1.34 (1.18-1.51) |  |  |
| Female | -0.21 (-0.36--0.05) | 1 | 0.0091 |
| Weight (natural spline basis 1) | -1.76 (-2.08--1.44) | 2 | < 0.0001 |
| Weight (natural spline basis 2) | -0.35 (-0.51--0.18) |  |  |
|  |  |  |  |
| Adjustment Model: | age*, sex, and weight** or breed*** |  |  |
|  | **Beta (95% CI)** | **df** | **P value** |
| **Gastrointestinal** |  |  |  |
| Commercial Raw | 0.20 (0.01-0.38) | 1 | 0.0373* |
| Home-cooked | **0.36 (0.21-0.51)** | **1** | **< 0.0001** |
| Home-prepared Raw | -0.63 (-1.08--0.18) | 1 | 0.0064* |
| Age (natural spline basis 1) | 0.65 (0.46-0.83) | 2 | < 0.0001 |
| Age (natural spline basis 2) | 0.43 (0.35-0.52) |  |  |
| Female | -0.14 (-0.21--0.07) | 1 | 0.0002 |
| Weight among mixed-breed (natural spline basis 1) | 0.14 (-0.09-0.37) | 2 | 0.4704 |
| Weight among mixed-breed (natural spline basis 2) | 0.05 (-0.08-0.17) |  |  |
| **Renal/Urinary** |  |  |  |
| Commercial Raw | -0.19 (-0.44-0.06) | 1 | 0.1372 |
| Home-cooked | **0.30 (0.12-0.47)** | **1** | **0.0012** |
| Home-prepared Raw | -0.02 (-0.42-0.39) | 1 | 0.9295 |
| Age (natural spline basis 1) | 1.70 (1.43-1.96) | 2 | < 0.0001 |
| Age (natural spline basis 2) | 1.26 (1.16-1.36) |  |  |
| Female | 1.10 (1.00-1.21) | 1 | < 0.0001 |
| Weight among mixed-breed (natural spline basis 1) | 0.34 (0.04-0.63) | 2 | 0.0799 |
| Weight among mixed-breed (natural spline basis 2) | 0.05 (-0.13-0.22) |  |  |
| **Tumor** |  |  |  |
| Commercial Raw | 0.00 (-0.25-0.25) | 1 | 0.9945 |
| Home-cooked | 0.20 (0.01-0.39) | 1 | 0.0359* |
| Home-prepared Raw | 0.40 (0.06-0.74) | 1 | 0.0223* |
| Age (natural spline basis 1) | 4.65 (4.17-5.13) | 2 | < 0.0001 |
| Age (natural spline basis 2) | 2.30 (2.16-2.44) |  |  |
| Female | -0.02 (-0.11-0.07) | 1 | 0.7198 |
| Weight among mixed-breed (natural spline basis 1) | 1.58 (1.24-1.92) | 2 | < 0.0001 |
| Weight among mixed-breed (natural spline basis 2) | 0.66 (0.52-0.80) |  |  |
| **Cardiac** |  |  |  |
| Commercial Raw | 0.22 (0.00-0.45) | 1 | 0.0474* |
| Home-cooked | 0.19 (-0.00-0.38) | 1 | 0.0547 |
| Home-prepared Raw | -0.21 (-0.76-0.34) | 1 | 0.4521 |
| Age (natural spline basis 1) | 3.61 (3.13-4.08) | 2 | < 0.0001 |
| Age (natural spline basis 2) | 2.01 (1.86-2.15) |  |  |
| Female | -0.12 (-0.23--0.02) | 1 | 0.0219 |
| Weight among mixed-breed (natural spline basis 1) | -2.01 (-2.30--1.72) | 2 | < 0.0001 |
| Weight among mixed-breed (natural spline basis 2) | -0.69 (-0.93--0.45) |  |  |
| **Brain/Neurologic** |  |  |  |
| Commercial Raw | 0.12 (-0.20-0.43) | 1 | 0.4754 |
| Home-cooked | 0.15 (-0.09-0.40) | 1 | 0.221 |
| Home-prepared Raw | -0.67 (-1.48-0.13) | 1 | 0.1003 |
| Age (natural spline basis 1) | 3.23 (2.76-3.71) | 2 | < 0.0001 |
| Age (natural spline basis 2) | 1.84 (1.68-2.01) |  |  |
| Female | -0.31 (-0.44--0.18) | 1 | < 0.0001 |
| Weight among mixed-breed (natural spline basis 1) | -0.20 (-0.60-0.21) | 2 | 0.329 |
| Weight among mixed-breed (natural spline basis 2) | 0.09 (-0.13-0.31) |  |  |
| **Endocrine** |  |  |  |
| Commercial Raw | 0.31 (0.00-0.61) | 1 | 0.0499* |
| Home-cooked | 0.21 (-0.05-0.48) | 1 | 0.1105 |
| Home-prepared Raw | 0.21 (-0.35-0.77) | 1 | 0.4627 |
| Age (natural spline basis 1) | 5.41 (4.64-6.17) | 2 | < 0.0001 |
| Age (natural spline basis 2) | 2.49 (2.26-2.72) |  |  |
| Female | -0.11 (-0.24-0.02) | 1 | 0.1003 |
| Weight among mixed-breed (natural spline basis 1) | 0.88 (0.46-1.31) | 2 | < 0.0001 |
| Weight among mixed-breed (natural spline basis 2) | 0.54 (0.33-0.74) |  |  |
| **Hepatic/Pancreatic** |  |  |  |
| Commercial Raw | 0.06 (-0.28-0.39) | 1 | 0.732 |
| Home-cooked | **0.44 (0.21-0.67)** | **1** | **0.0002** |
| Home-prepared Raw | -0.08 (-0.75-0.59) | 1 | 0.8122 |
| Age (natural spline basis 1) | 3.58 (3.00-4.15) | 2 | < 0.0001 |
| Age (natural spline basis 2) | 1.80 (1.62-1.98) |  |  |
| Female | 0.11 (-0.03-0.24) | 1 | 0.1373 |
| Weight among mixed-breed (natural spline basis 1) | -0.85 (-1.26--0.44) | 2 | 0.0001 |
| Weight among mixed-breed (natural spline basis 2) | -0.37 (-0.64--0.09) |  |  |

Supplementary Table 3. Logistic regression results for pre-specified analytic models for all 13 health conditions. *Age was modeled using natural splines with knots at 2, 7, and 13 years **Weight was modeled using natural splines with knots at 15, 52, and 85 lbs. ***Models were fit with conditional logistic regression which do not provide estimates for breed effects.
